# Retraction: The fabrication of a chemical sensor with PANI-TiO_2_ nanocomposites

**DOI:** 10.1039/d5ra90145f

**Published:** 2026-01-12

**Authors:** Mohammad R. Karim, M. M. Alam, M. O. Aijaz, Abdullah M. Asiri, F. S. AlMubaddel, Mohammed M. Rahman

**Affiliations:** a Center of Excellence for Research in Engineering Materials (CEREM), King Saud University Riyadh 11421 Saudi Arabia; b K. A. CARE Energy Research and Innovation Center Riyadh 11451 Saudi Arabia; c Department of Chemical Engineering and Polymer Science, Shahjalal University of Science and Technology Sylhet 3100 Bangladesh alam-mahmud@hotmail.com; d Center of Excellence for Advanced Materials Research & Chemistry Department, Faculty of Science, King Abdulaziz University Jeddah 21589 Saudi Arabia; e Chemical Engineering Department, King Saud University Riyadh 11421 Saudi Arabia

## Abstract

Retraction of “The fabrication of a chemical sensor with PANI-TiO_2_ nanocomposites” by Mohammad R. Karim *et al.*, *RSC Adv.*, 2020, **10**, 12224–12233, https://doi.org/10.1039/C9RA09315J.

The Royal Society of Chemistry hereby wholly retracts this *RSC Advances* article due to evidence that the peer review process was manipulated.

An investigation has established that the acceptance of this article was based on a fake reviewer report. The named reviewer does not have access to the email address used and they confirmed that they did not submit the report. Our investigation has found that the report was written and submitted by the last author. We have therefore concluded that the peer review process for this paper was compromised.

The other authors were not aware of, did not participate in, and did not authorise any irregularities in the peer review process. All co-authors were informed of the decision to retract this article. M. M. Alam did not respond to any correspondence and the other authors disagree with the decision to retract.

Signed: Laura Fisher, Executive Editor, *RSC Advances*

Date: 19th December 2025

